# Neuroprotection by Cocktails of Dietary Antioxidants under Conditions of Nerve Growth Factor Deprivation

**DOI:** 10.1155/2015/217258

**Published:** 2015-07-08

**Authors:** Flavio Amara, Miluscia Berbenni, Martina Fragni, Giampaolo Leoni, Sandra Viggiani, Vita Maria Ippolito, Marilena Larocca, Rocco Rossano, Lilia Alberghina, Paolo Riccio, Anna Maria Colangelo

**Affiliations:** ^1^Laboratory of Neuroscience “R. Levi-Montalcini”, Department of Biotechnology and Biosciences, University of Milano-Bicocca, 20126 Milan, Italy; ^2^SYSBIO Centre of Systems Biology, University of Milano-Bicocca, 20126 Milan, Italy; ^3^Department of Sciences, University of Basilicata, 85100 Potenza, Italy; ^4^NeuroMI Milan Center for Neuroscience, University of Milano-Bicocca, 20126 Milan, Italy

## Abstract

Dietary antioxidants may be useful in counteracting the chronic inflammatory status in neurodegenerative diseases by reducing oxidative stress due to accumulation of reactive oxygen species (ROS). In this study, we newly described the efficacy of a number of dietary antioxidants (polyphenols, carotenoids, thiolic compounds, and oligoelements) on viability of neuronal PC12 cells following Nerve Growth Factor (NGF) deprivation, a model of age-related decrease of neurotrophic support that triggers neuronal loss. Neuroprotection by antioxidants during NGF deprivation for 24 h was largely dependent on their concentrations: all dietary antioxidants were able to efficiently support cell viability by reducing ROS levels and restoring mitochondrial function, while preserving the neuronal morphology. Moreover, ROS reduction and neuroprotection during NGF withdrawal were also achieved with defined cocktails of 3–6 different antioxidants at concentrations 5–60 times lower than those used in single treatments, suggesting that their antioxidant activity was preserved also at very low concentrations. Overall, these data indicate the beneficial effects of antioxidants against oxidative stress induced by decreased NGF availability and suggest that defined cocktails of dietary factors at low concentrations might be a suitable strategy to reduce oxidative damage in neurodegenerative diseases, while limiting possible side effects.

## 1. Introduction

Oxidative stress and mitochondrial dysfunction are common outcomes of inflammatory conditions which have been involved in the pathogenesis of chronic neurodegenerative disorders, including Alzheimer's disease (AD), Parkinson's disease (PD), Multiple Sclerosis (MS), and Amyotrophic Lateral Sclerosis (ALS) [1–3]. Oxidative stress can be induced by environmental toxins (MPTP and pesticides in PD) [4], poor dietary habits and gut dysbiosis, excitotoxicity (particularly relevant in ALS), and several age-related alterations such as accumulation of amyloid-beta (A*β*) and reduced neurotrophic support in AD [5]. In particular, age-dependent decrease of Nerve Growth Factor (NGF) [5–7] has been functionally linked to sporadic forms of AD because of its role in regulating survival and synaptic plasticity of basal forebrain cholinergic neurons [8]. Moreover, NGF deficiency in AD11 transgenic mice has been found to determine the appearance of all AD hallmarks, including A*β* plaques, hyperphosphorylated tau tangles, loss of cholinergic neurons, and cognitive dysfunction [9].

Molecular mechanisms of apoptotic death triggered by decreased NGF availability have been largely elucidated in PC12 cells and primary neurons [5]; they involve oxidative stress and mitochondrial dysfunction [10, 11], due to the role of NGF in regulating the balance of proapoptotic and antiapoptotic bcl-2 family proteins [12] and regulation of antioxidant enzymes through the PI3K/Akt and NF-*κ*B signaling [13–15]. Neurons are particularly susceptible to oxidative damage due to their dependence on oxidative phosphorylation for their large energy demand. Increased oxidative stress, due to accumulation of reactive oxygen (ROS) and nitrogen (RNS) species, is the result of an imbalance between generation of free radicals and endogenous antioxidant defenses. The increase of free radicals causes oxidative damage to mitochondrial proteins, lipids, and DNA and leads to decreased mitochondrial membrane potential and ATP depletion.

Several epidemiological studies have shown that diets rich in vegetables, fruit, fiber, and fish are relevant to health and longevity and protect against cancer and cardiovascular and neurodegenerative diseases, whereas diets based on consumption of saturated fatty acids of animal origin, transfatty acids, simple sugars, and red meat favor a chronic inflammatory status [16–18]. Many “healthy” dietary factors present in a vegetarian diet are known for their antioxidant properties, but it is now established that they have additional biological properties going far beyond their simple antioxidant activity. For instance, they are able to modulate cell metabolism and reduce inflammation by interacting with enzymes, nuclear receptors, and transcription factors [16].

Natural antioxidants and anti-inflammatory compounds include polyphenols, carotenoids, thiolic compounds, and oligoelements as selenium.

Polyphenols (present in vegetables, spices, herbs, fruits, and wine) include flavonoids and nonflavonoids molecules [19, 20]. The most important flavonoids are quercetin (QRC) and catechins (green tea extract, GTE). The most important nonflavonoids molecules are resveratrol (RSV), curcumin (CRC), and hydroxytyrosol (in this study present in Oliplus, OLP). Polyphenols are not well known molecules with regard to their bioavailability and biological effects, but all of them are able to counteract the negative effects of microbial agents and/or saturated- or transfatty acids, downregulating the expression of proinflammatory molecules, oxidative stress, and angiogenesis.

QRC is present mainly as a glucoside and its effects may be additive to those of interferon-*β*. QRC is not toxic, but its oxidation product (quercetin quinone) is very reactive towards -SH groups of proteins and glutathione and may be toxic [21]. Addition of thiolic compounds such as *α*-lipoic acid (ALA) or N-acetylcysteine (NAC) can limit the toxic effects. RSV is glucuronated in the liver and absorbed as such in limited amount mainly in the duodenum. Its glucuronidation is inhibited by QRC [22]. RSV is a typical molecule with hormesis effects on a wide variety of cells [23]: its neuroprotective activity is well known [24–26]; however, depending on its concentration, it can also induce apoptosis [22]. These discrepancies can be attributed to the different concentrations used* in vitro* or bioavailable* in vivo*, since RSV appears to have opposite effects at concentrations of 10^−5 ^M (proliferation of human mesenchymal cells) and 10^−4 ^M (inhibition of proliferation) [27, 28]. In our experience, RSV has a neurotrophic effect on cortical neurons in culture only at very low concentrations, whereas at higher concentration RSV may have toxic effects (Colangelo, AM, unpublished).

Based on these considerations, and due to their metabolic relevance and their limited bioavailability, polyphenols may be recommended as a mixture of different flavonoids and nonflavonoids, since it has been shown that their properties may depend on their specific chemical structure [29]. The combinatorial treatment with polyphenols and other antioxidant agents at low concentration may be an appropriate strategy to increase the bioavailability of dietary molecules and prevent their metabolic transformations or unwanted toxic effects, while providing effective neuroprotection against oxidative stress.

Other natural antioxidants are carotenoids, thiolic compounds, and selenium. Among carotenoids, the most important is lycopene (LYC) (present in tomato, water melon, and pink grape fruit) [30]. Compounds containing thiol groups (-SH), such as *α*-lipoic acid (ALA), glutathione, and N-acetylcysteine (NAC), are important dietary supplements for the complementary treatment of chronic inflammatory diseases. ALA (green plants and animal foods) has immunomodulatory and anti-inflammatory properties: it stimulates protein kinase A and cAMP production and inhibits the synthesis of interferon-*γ* and adhesion molecules [31]. Moreover, ALA has been found to be effective in the Experimental Autoimmune Encephalomyelitis (EAE) model of MS and stabilizes the integrity of the blood-brain barrier (BBB) [32, 33]. NAC also passes through the BBB and protects cardiac and neuronal tissues from inflammation and oxidative stress [34, 35]. Among oligoelements, selenium regulates the redox status of vitamin C and protects against oxidative stress in chronic hypertension, cardiovascular disease, cancer, and aging [36].

A large number of studies support the beneficial effects of antioxidants in several models of neuronal injury, both* in vitro* and* in vivo* [24–26, 37, 38]. However, the neuroprotective activity of antioxidants against NGF deficiency has never been reported so far. Therefore, in this study, we have used neuronal NGF-dependent PC12 cells to analyze the antioxidant properties of several natural antioxidant molecules during NGF deprivation. Our data indicate that all antioxidants protect neuronal cells during NGF withdrawal by reducing ROS levels and mitochondrial dysfunction. Moreover, the neuroprotective activity showed by defined cocktails of dietary factors at low concentrations suggests a suitable strategy to reduce oxidative damage in neurodegenerative diseases, while limiting possible side effects.

## 2. Materials and Methods

### 2.1. Chemicals

Murine 2.5S NGF (mNGF) purified from male mouse submaxillary glands was purchased from Promega Inc. (Madison WI, USA). Resveratrol (RSV), quercetin (QRC), curcumin (CRC), lycopene (LYC), alpha-lipoic acid (ALA), Oliplus (OLP, a mixture containing hydroxytyrosol), green tea extract (GTE), and N-acetylcysteine (NAC) were from Nutraceutica srl (Monterenzio, Bologna, Italy). Acetyl-L-Carnitine (ALCAR), Coenzyme Q10 (CoQ), and selenium (Sel) were purchased from Sigma-Aldrich. *β*-III tubulin antibody was from Millipore. MitoTracker Red/Green and 2′,7′-dichlorofluorescein-diacetate (DCFH-DA) were purchased from Molecular Probes (Eugene, OR).

### 2.2. Cell Cultures and Treatments

PC12 cells (*clone 615*) [39] overexpressing the TrkA NGF receptor [40] (kindly provided by MV Chao, Skirball Institute of Biomolecular Medicine, New York University School of Medicine, NY) were maintained in Dulbecco's modified Eagle medium (DMEM, Sigma-Aldrich) supplemented with 10% fetal bovine serum (FBS, Sigma-Aldrich), 5% heat-inactivated horse serum (HS, Sigma-Aldrich), 2 mM L-glutamine, 100 *μ*g/mL streptomycin, 100 U/mL penicillin, and 200 *μ*g/mL G418 (Sigma-Aldrich), in a humidified atmosphere of 95% air 5% CO_2_ at 37°C, as previously described [9].

All experiments were performed on fully differentiated PC12(615) cells following 6-day exposure to 2.5S mNGF (10 ng/mL, Promega Inc., Madison WI, USA) in low-serum DMEM supplemented with 1% FBS and 0.5% heat-inactivated HS. Medium containing NGF was changed every other day and immediately before treatments, which were performed in low-serum media. In all experiments, NGF-differentiated cells were pretreated overnight with selected antioxidants before exposure to NGF-free medium in the presence of the antioxidants. For dose-response studies, antioxidant molecules were used at the following concentrations: RSV 1–100 *μ*M, QRC 1–100 *μ*M, CRC 1–100 *μ*M, OLP 1–500 *μ*g/mL, GTE 12.5–100 *μ*g/mL, LYC 1–50 *μ*M, ALA 1–100 *μ*M, ALCAR 1–100 *μ*M, NAC 5–300 *μ*M, CoQ 0.1–100 *μ*M, and Sel 0.5-100 nM. Composition of defined cocktails of antioxidants is shown in Table 1.

### 2.3. Cell Viability

Cell survival was assessed by the MTT assay based on reduction of the yellow tetrazolium salts (MTT) to the purple formazan by mitochondrial dehydrogenases. The assay was performed according to the specifications of the manufacturer (Sigma-Aldrich). Briefly, PC12 cells (5000 cells/well) were grown in 96-well plates (Euroclone) precoated with poly-L-lysine (1 mg/mL) and differentiated for 6 days in low-serum medium containing NGF (10 ng/mL). To analyze the effect of antioxidants on cell viability, NGF-differentiated cells were pretreated overnight with selected antioxidants and then changed for 24 h to NGF-free medium containing the antioxidant. Following treatments, tetrazolium salts (0.5 mg/mL) were added directly to the culture medium for 4 hr in a humidified atmosphere. After incubation, 100 *μ*L of the MTT solubilization solution were added to each well for 1 hr. Absorbance of samples was measured at 570 nm (700 nm reference wavelength) with a Microplate Reader (BioRad, Hercules, CA). MTT conversion levels were expressed as a percentage of control.

### 2.4. Determination of ROS

Intracellular ROS levels were measured as previously described [10] by using the fluorescent probe 2′,7′-dichlorodihydrofluorescein-diacetate (DCFH-DA; Molecular Probes, Eugene, OR). DCFH-DA is cleaved by cellular esterases to the nonfluorescent DCFH, which is oxidized to the highly fluorescent compound, 2′,7′-dichlorofluorescein (DCF). In brief, PC12 cells (10^5^ cells/well) were grown onto 6-well plates (Euroclone) precoated with poly-L-lysine (1 mg/mL) and differentiated for 6 days with mNGF (10 ng/mL). Cells were preincubated overnight with the specific antioxidants followed by exposure to NGF-free medium for 6 h. DCFH-DA (10 *μ*M) was added during the last 30 min of treatments. Cells were then washed with PBS, harvested in 0.25% trypsin, and analyzed by FACS (FACScan, Becton-Dickinson, San Jose, CA), using the Cell Quest software (BD Bioscience). Flow cytometric measurements (Geo-mean values) were taken on 10,000 cells contained in the gated regions. Data analysis was performed with WinMDI software. For fluorescence microscopy analysis, cells (2 × 10^4^) were grown onto 12 mm poly-L-lysine-coated coverslips and differentiated with mNGF (10 ng/mL). DCFH-DA (10 *μ*M) was added during the last 30 min of treatments. Cells were counterstained with 4,6-diamidino-2-phenylindole (DAPI) for 1 min and rinsed three times with PBS. Coverslips were mounted with ProLong Gold Antifade reagent (Molecular Probes, Eugene, OR) and analyzed by fluorescence microscopy. Cells were imaged at 40x magnification using a motorized Nikon Eclipse 90i (Nikon, Tokyo, Japan) fluorescence microscope equipped with a CCD camera (Hamamatsu-CoolSnap, Hamamatsu Corporation, Tokyo, Japan). NIH-ImageJ software was used for image analysis and processing. The mean fluorescence intensity (MFI = fluorescence intensity divided by the total number of cells) was calculated on about 150 cells in about 10 random fields for each condition.

### 2.5. Analysis of Mitochondrial Function

Mitochondrial potential was assessed as previously described [10] by using MitoTracker Red (CMXRos) and Green (Molecular Probes, Eugene, OR) staining, indicators of mitochondrial potential and mass/morphology, respectively. Briefly, cells (2 × 10^4^) were plated onto 12 mm poly-L-lysine-coated coverslips and differentiated for 6 days with mNGF (10 ng/mL). Cells were pretreated overnight with the specific antioxidants and then switched to NGF-free medium for 12 h. Cells were loaded with 50 and 200 nM MitoTracker Red and Green, respectively, during the last 30 min of treatment and then rinsed twice with PBS. Coverslips were mounted with ProLong Gold Antifade reagent (Molecular Probes, Eugene, OR) and analyzed by fluorescence microscopy. Images were captured at 60x magnification (Nikon Plan Apo 60x oil objective) using a motorized Nikon Eclipse 90i (Nikon, Tokyo, Japan) fluorescence microscope equipped with a CCD camera (Hamamatsu-CoolSnap, Hamamatsu Corporation, Tokyo, Japan). NIH-ImageJ software was used for image analysis and processing.

### 2.6. Immunocytochemistry

Cells (2 × 10^4^) were grown onto 12 mm poly-L-lysine coated coverslips and differentiated for 6 days with mNGF (10 ng/mL). Cells were then fixed in 4% paraformaldehyde for 10 min and permeabilized with 0.1% Triton X-100 in PBS for 15 min. Nonspecific binding was blocked by incubation in PBS containing 1% bovine serum albumin (BSA) and 10% normal goat serum for 30 min. Cells were then incubated with mouse anti-*β*-III tubulin antibody (Millipore; 1 : 500) in PBS/1% BSA/10% goat serum for 1 hr at room temperature. After three washes in PBS, cells were incubated with goat anti-mouse Alexa 488-conjugated antibody (Molecular Probes; 1 : 200) in PBS/1% BSA/10% goat serum for 1 hr at room temperature, followed by counterstaining with DAPI for 1 min. After rinsing in PBS, coverslips were mounted with ProLong Gold Antifade reagent (Molecular Probes). Images were acquired at 40x magnification using a motorized Nikon Eclipse 90i (Nikon, Tokyo, Japan) fluorescence microscope equipped with a CCD camera (Hamamatsu-CoolSnap, Hamamatsu Corporation, Tokyo, Japan). NIH-ImageJ software was used for image analysis and processing.

### 2.7. Statistical Analysis

All data were represented as the mean ± SEM. Statistical analysis was performed by using GraphPad Prism for Windows, version 6.0 (GraphPad Software, San Diego, CA, USA). Differences between groups were determined by ANOVA and Dunnett's multiple comparisons test. Values of *p* < 0.05 or < 0.01 or < 0.001 were considered as statistically significant.

## 3. Results

### 3.1. Neuroprotection by Antioxidant Molecules following NGF Withdrawal

**Figure 1:**
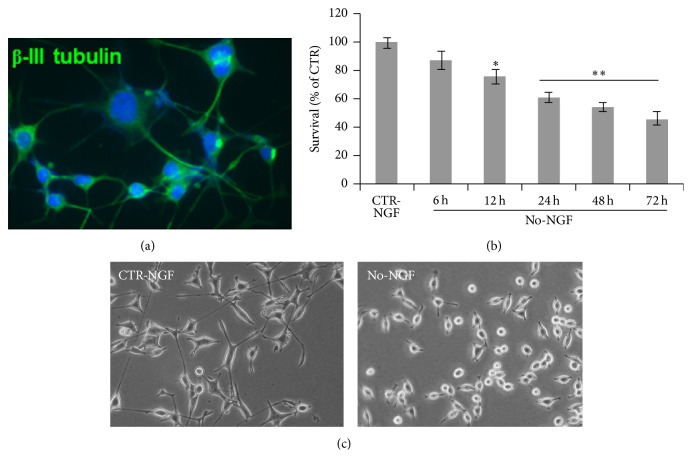
Time-course of neuronal PC12 cells death following NGF deprivation. (a) *β*-III tubulin immunostaining on PC12(615) cells differentiated with NGF (10 ng/mL) for 6 days. (b) MTT assay on neuronal PC12 cells NGF-deprived for the indicated times. Data, expressed as percent of CTR-NGF at the indicated time points, are the mean ± SEM of five independent experiments, each performed in triplicate. CTR-NGF did not change at each time point. ^*∗*^ *p* ≤ 0.05, ^*∗∗*^ *p* ≤ 0.01 versus CTR-NGF (ANOVA and Dunnett's multiple comparisons test). (c) Representative images of neuronal PC12 cells, CTR, or NGF-deprived for 72 h.

Antioxidants exert neuroprotection in several models of neuronal injury, both* in vitro* and* in vivo* [24–26]. However, their capability to protect neurons under conditions of decreased neurotrophic support has not been previously investigated. To examine whether antioxidant molecules are able to preserve neuronal survival following reduced neurotrophic support, we used NGF-differentiated PC12 cells (clone 615) [40]. Upon exposure to NGF (10 ng/mL) for 6 days, PC12 cells differentiate into sympathetic-like neurons and become NGF-dependent [10]. NGF-differentiated cells, which expressed the neuronal-specific *β*-III-tubulin (Figure 1(a)), were changed to NGF-free medium for 6 to 72 h and cell viability was assessed by MTT assay.  Figure 1(b) shows that NGF withdrawal caused a significant time-dependent decrease of survival by 12–24 h, with most cells losing their neurite processes and showing a round-shaped morphology by 72 h (Figure 1(c)).

**Figure 2:**
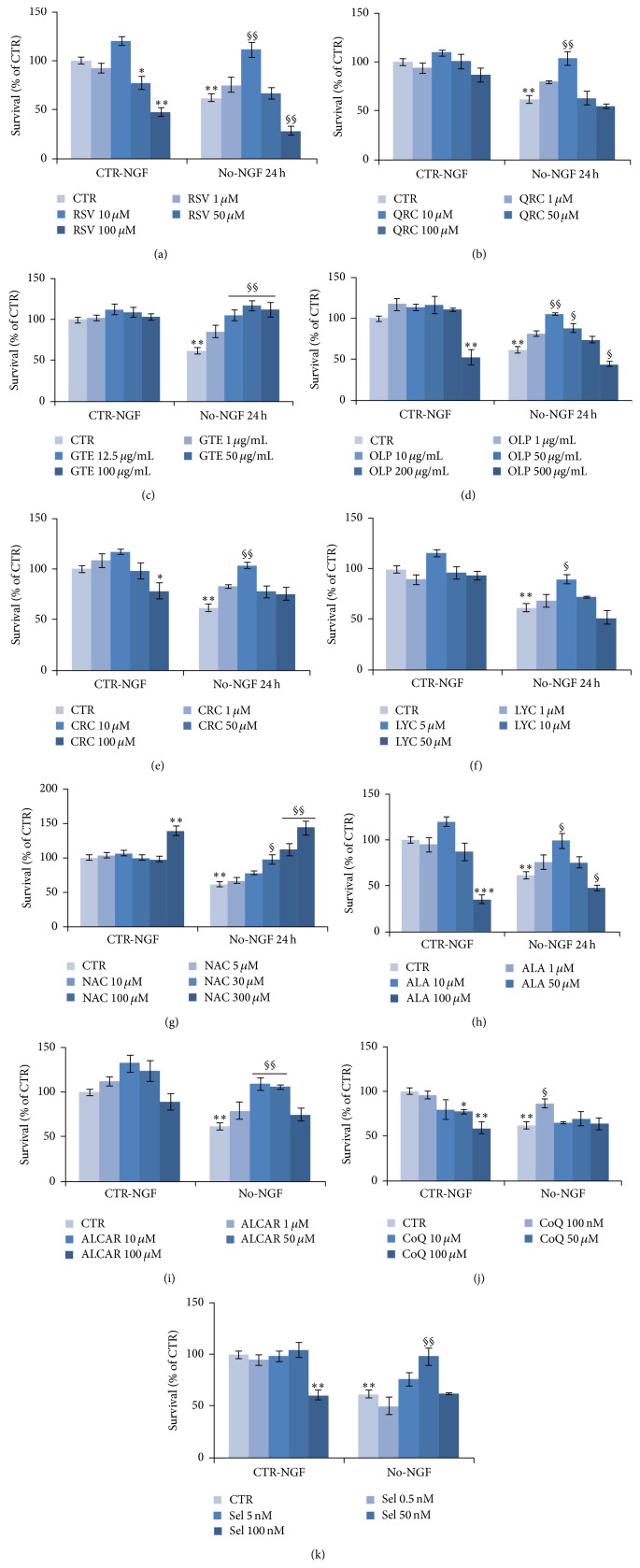
Neuroprotection by antioxidant molecules against NGF deprivation. MTT assay on neuronal PC12 cells exposed to NGF deprivation for 24 h. Where indicated, cells were preincubated overnight with RSV (a), QRC (b), GTE (c), OLP (d), CRC (e), LYC (f), NAC (g), ALA (h), ALCAR (i), CoQ (j), or Sel (k) at the indicated concentrations followed by NGF deprivation for 24 h in the presence of the antioxidants. Data are the mean ± SEM of three experiments, each performed in triplicate. ^*∗*^ *p* ≤ 0.05, ^*∗∗*^ *p* ≤ 0.01, and ^*∗∗∗*^ *p* ≤ 0.001 versus CTR-NGF; ^§^ *p* ≤ 0.05, ^§§^ *p* ≤ 0.01 versus No-NGF (ANOVA and Dunnett's multiple comparisons test).

**Figure 3:**
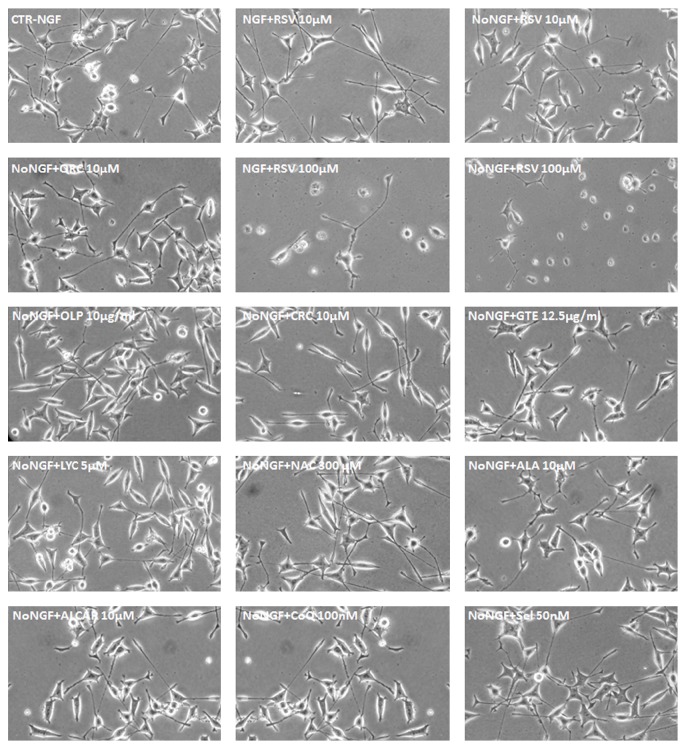
Morphology of neuronal PC12 cells during NGF deprivation in the presence with antioxidants. Representative images of neuronal PC12 cells maintained in the presence of NGF (CTR-NGF) or NGF-deprived for 24 h (No-NGF) or exposed to NGF-free medium containing the indicated antioxidants (RSV 10–100 *μ*M, QRC 10 *μ*M, OLP 10 *μ*g/mL, CRC 10 *μ*M, GTE 12.5 *μ*g/mL, LYC 5 *μ*M, NAC 300 *μ*M, ALA 10 *μ*M, ALCAR 10 *μ*M, CoQ 100 nM, and Sel 50 nM).

We then used neuronal PC12 cells to evaluate neuroprotection during NGF deprivation for 24 h by a number of antioxidants, including flavonoids (QRC, GTE), nonflavonoids (RSV, CRC, and OLP), carotenoids (LYC), thiol compound (NAC, ALA), ALCAR, CoQ, and Sel. To this purpose, neuronal NGF-dependent PC12 cells were pretreated overnight with distinct antioxidants (RSV, QRC, CRC, OLP, GTE, LYC, NAC, ALA, ALCAR, CoQ, or Sel) and then switched for 24 h to NGF-free medium containing the specific antioxidant. Different concentrations were tested for each molecule, because of the discrepancy between effective doses reported in distinct cellular types. In agreement with previous studies on other models of neuronal injury [4, 41, 42], MTT assay revealed that pretreatment of neuronal PC12 cells with RSV (10 *μ*M) significantly improved cell viability both under basal conditions (CTR-NGF) and following NGF withdrawal (No-NGF) for 24 h (Figure 2(a)). Instead, lower (1 *μ*M) or higher (50–100 *μ*M) concentrations either were ineffective or dramatically decreased cell viability even in the presence of NGF (Figure 2(a)). It was also important to observe that pretreatment with RSV 10 *μ*M preserved the neuron-like morphology of neuronal PC12 cells during NGF deprivation for 24 h (Figure 3), while extensive cell loss was evident when cells were maintained in the presence of RSV 100 *μ*M, as compared to the CTR (Figure 3).

A similar trend was found for most antioxidant molecules, as their best neuroprotective activity occurred at low concentrations. Thus, effective neuroprotection during NGF withdrawal was achieved when NGF-differentiated cells were pretreated with QRC 10 *μ*M (Figure 2(b)), OLP 10 *μ*g/mL (Figure 2(d)), CRC 10 *μ*M (Figure 2(e)), LYC 5 *μ*M (Figure 2(f)), ALA 10 *μ*M (Figure 2(h)), ALCAR 10–50 *μ*M (Figure 2(i)), CoQ 100 nM (Figure 2(j)), or Sel 50 nM (Figure 2(k)). Instead, higher doses of these antioxidants were unable to protect neuronal PC12 cells during NGF deprivation and significantly decreased survival even under control conditions (CTR-NGF), as shown by OLP 500 *μ*g/mL (Figure 2(d)), CRC 100 *μ*M (Figure 2(e)), ALA 100 *μ*M (Figure 2(h)), CoQ 10–100 *μ*M (Figure 2(j)), and Sel 100 nM (Figure 2(k)). GTE and NAC supported survival at all concentrations, but a strong increase of cell viability was found with GTE 12.5–100 *μ*g/mL (Figure 2(c)) or NAC 300 *μ*M (Figure 2(g)). All molecules at their effective concentrations were also able to sustain the maintenance of neuronal PC12 cells morphology during NGF withdrawal (Figure 3).

### 3.2. Antioxidants Prevented Intracellular ROS Increase and Mitochondrial Dysfunction following NGF Deprivation

**Figure 4:**
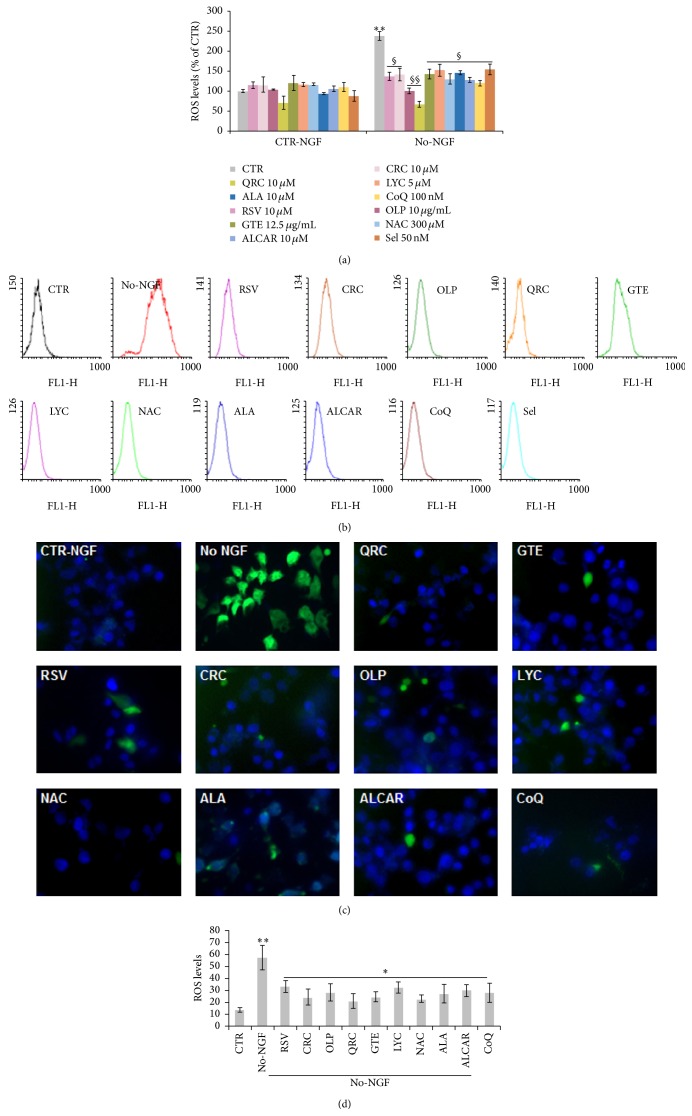
Effect of antioxidants on ROS levels following NGF deprivation. (a) Quantitation of ROS levels by FACS analysis of DCHF-DA fluorescence. Neuronal PC12 cells were preincubated overnight with RSV (10 *μ*M), CRC (10 *μ*M), OLP (10 *μ*g/mL), QRC (10 *μ*M), GTE (12.5 *μ*g/mL), LYC (5 *μ*M), NAC (300 *μ*M), ALA (10 *μ*M), ALCAR (10 *μ*M), CoQ (100 nM), or Sel (50 nM) and then changed for 6 h to NGF-free medium containing the same antioxidant. Cells were loaded with DCFH-DA (10 *μ*M) during the last 30 min of treatments and flow cytometric measurements (Geo-mean values) were taken on 10,000 cells contained in the gated regions. Data are the mean ± SEM of three experiments in duplicate. ^*∗∗*^ *p* ≤ 0.01 versus CTR-NGF; ^§^ *p* ≤ 0.05, ^§§^ *p* ≤ 0.01 versus No-NGF (ANOVA and Dunnett's multiple comparisons test). (b) FACS profiles of a representative experiment. (c) Representative images of fluorescence microscopy analysis of ROS levels on neuronal PC12 cells after NGF deprivation for 6 h in the presence of RSV (10 *μ*M), CRC (10 *μ*M), OLP (10 *μ*g/mL), QRC (10 *μ*M), GTE (12.5 *μ*g/mL), LYC (5 *μ*M), NAC (300 *μ*M), ALA (10 *μ*M), ALCAR (10 *μ*M), or CoQ (100 nM). Cells were loaded with DCFH-DA (10 *μ*M) during the last 30 min of treatments and observed under a fluorescence microscope (Nikon) equipped with a CCD camera. Images were captured at 40x magnification. Scale bar = 10 *μ*m. (d) Quantitation of DCHF-DA positive cells by NIH ImageJ. MFI was calculated on about 150 cells in 10 random fields for each condition. Data are the mean ± SEM of three experiments in duplicate. ^*∗*^ *p* ≤ 0.05 versus No-NGF, ^*∗∗*^ *p* ≤ 0.01 versus CTR (ANOVA and Dunnett's multiple comparisons test).

Accumulation of ROS is known to occur in response to many toxic insults, including decreased neurotrophic support [10, 43]. To assess the antioxidant activity of all dietary molecules, we measured intracellular ROS levels in neuronal PC12 cells by using the oxidation-sensitive fluorescent dye DCHF-DA (Molecular Probes). Flow cytometry analysis of NGF-differentiated PC12 cells maintained in the presence of NGF showed low levels of DCFH-DA fluorescence. As reported in our previous studies [10], ROS levels were significantly enhanced by about 2.5-fold when neuronal PC12 cells were exposed to NGF-free medium for 6 h (Figure 4(a)), as also shown by FACS analysis profiles in Figure 4(b).

To examine whether antioxidants could prevent the increase of ROS induced by NGF deprivation, neuronal PC12 cells were pretreated overnight with selected antioxidants and then switched to NGF-free medium for 6 h.  Figure 4 shows that the increase of ROS levels was significantly reduced by overnight preincubation with RSV (10 *μ*M), CRC (10 *μ*M), OLP (10 *μ*g/mL), QRC (10 *μ*M), GTE (12.5 *μ*g/mL), LYC (5 *μ*M), NAC (300 *μ*M), ALA (10 *μ*M), ALCAR (10 *μ*M), CoQ (100 nM), or Sel (50 nM) (Figures 4(a)-4(b)), before NGF withdrawal. The effect of antioxidants on intracellular ROS levels was also evident by fluorescence microscopy analysis, as shown in Figures 4(c)-4(d).

**Figure 5:**
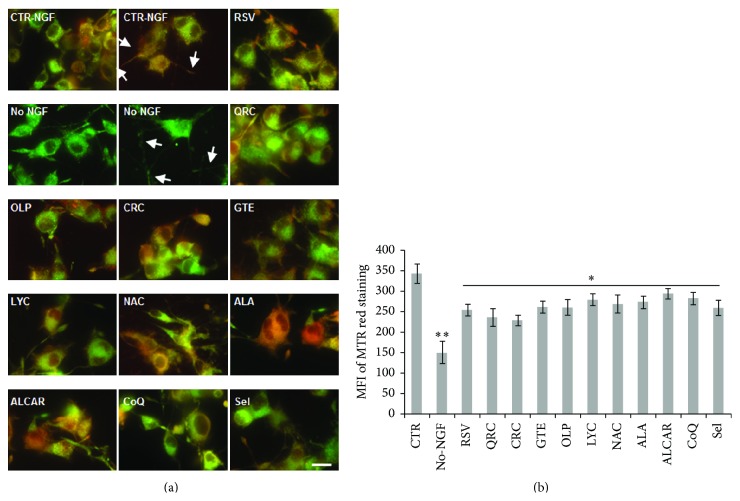
Analysis of mitochondrial function by MitoTracker Red/Green staining. (a) Representative merged images of neuronal PC12 cells maintained in the presence of NGF (CTR-NGF), NGF-deprived for 12 h (No-NGF), or exposed to NGF-free medium containing the antioxidants. NGF-differentiated PC12 cells were pretreated overnight with of RSV (10 *μ*M), QRC (10 *μ*M), OLP (10 *μ*g/mL), CRC (10 *μ*M), GTE (12.5 *μ*g/mL), LYC (5 *μ*M), NAC (300 *μ*M), ALA (10 *μ*M), ALCAR (10 *μ*M), CoQ (100 nM), or Sel (50 nM) before NGF withdrawal in the presence of the antioxidants. Cells were stained by MitoTracker Red and Green (50 and 200 nM, resp.) during the last 30 min of treatment and observed with a fluorescence microscope (Nikon) equipped with a CCD camera. Images were captured at 60x magnification. Arrowheads point to decreased ΔΨ*m* along neurites. Scale bar = 10 *μ*m. (b) MitoTracker Red fluorescence was quantified by NIH ImageJ, and the MFI was calculated by counting all cells (on average 200 cells) in about 20 random fields for each condition. Data, expressed as MFI, are the mean ± SEM of three experiments in duplicate. ^*∗*^ *p* ≤ 0.05 versus No-NGF, ^*∗∗*^ *p* ≤ 0.01 versus CTR (ANOVA and Dunnett's multiple comparisons test).

Dysfunctional mitochondria are the main source of ROS. To assess the role of mitochondria in oxidative stress following NGF withdrawal, we measured the mitochondrial potential (ΔΨ*m*) by MitoTracker Red/Green staining. Preliminary time-course studies (data not shown) revealed that NGF-differentiated PC12 cells exposed to NGF deprivation for 12 h displayed a 40–50% decrease of mitochondrial staining (Figures 5(a)-5(b)). To examine whether antioxidants could prevent this decline of mitochondrial function, NGF-differentiated PC12 cells were preincubated overnight with distinct antioxidants and then changed for 12 h to NGF-free medium containing the specific antioxidant molecule. In agreement with their effect on survival and ROS levels, we found that the reduction of ΔΨ*m* induced by NGF withdrawal for 12 h was prevented by overnight pretreatment with RSV (10 *μ*M), CRC (10 *μ*M), OLP (10 *μ*g/mL), QRC (10 *μ*M), GTE (12.5 *μ*g/mL), LYC (5 *μ*M), NAC (300 *μ*M), ALA (10 *μ*M), ALCAR (10 *μ*M), CoQ (100 nM), or Sel (50 nM) (Figures 5(a)-5(b)). These data indicate that the beneficial effect of antioxidants in neuroprotection involved their role in preserving mitochondrial function and cellular redox homeostasis.

### 3.3. Neuroprotection of Neuronal PC12 Cells by Defined Cocktails of Antioxidants

**Figure 6:**
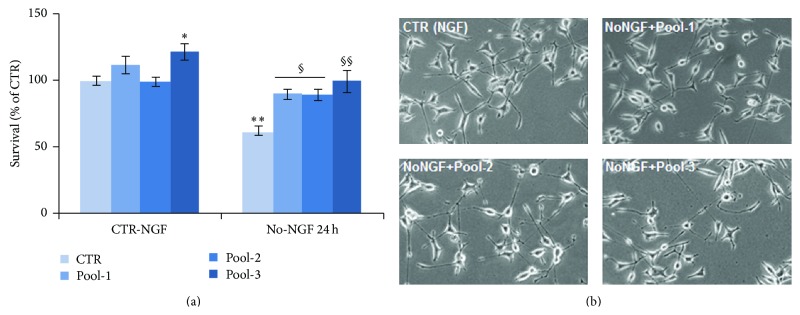
Neuroprotection by antioxidant cocktails against NGF deprivation. (a) MTT assay on neuronal PC12 cells exposed to NGF deprivation for 24 h. Where indicated, cells were NGF deprived following overnight preincubation with defined pools of antioxidant molecules (Pool-1, Pool-2, or Pool-3, see Table 1). Data are the mean ± SEM of three separate experiments, with three independent samples for each treatment. ^*∗*^ *p* ≤ 0.05, ^*∗∗*^ *p* ≤ 0.01 versus CTR-NGF; ^§^ *p* ≤ 0.05, ^§§^ *p* ≤ 0.01 versus No-NGF (ANOVA and Dunnett's multiple comparisons test). (b) Representative images of neuronal PC12 cells maintained in the presence of NGF or NGF-deprived for 24 h in the presence of the indicated antioxidant pools.

All antioxidant molecules have distinct modes of action. Moreover, on neuronal PC12 cells they showed their greatest neuroprotection at low concentrations and potential neurotoxicity at high doses. Based on these observations, we tested whether effective neuroprotection against NGF withdrawal could be achieved by defined cocktails of antioxidants, where antioxidant molecules were present at concentrations up to 5–60 times lower than those used in single treatments (Table 1). As shown in Figure 6, we found that overnight preincubation of neuronal PC12 cells with Pool-1 (RSV 2 *μ*M, QRC 2 *μ*M, NAC 5 *μ*M, and OLP 1 *μ*g/mL) was able to significantly prevent the reduction of neuronal viability induced by NGF withdrawal for 24 h (Figure 6(a)), as compared to the effect of single antioxidants at the same low doses used in the cocktail (Figure 2). Loss of survival induced by NGF deprivation was also prevented by overnight preincubation of neuronal PC12 cells with Pool-2 (RSV 1 *μ*M, QRC 1 *μ*M, NAC 10 *μ*M, OLP 1 *μ*g/mL, CRC 1 *μ*M, and LYC 1 *μ*M) and Pool-3 (RSV 2.5 *μ*M, ALA 2.5 *μ*M, and ALCAR 2.5 *μ*M) before switching cells to NGF-free medium for 24 h (Figure 6(a)), as compared to each single treatment (Figure 2). Survival of NGF-deprived PC12 cells was paralleled by the effect of Pool-1, Pool-2, and Pool-3 on maintenance of neuronal morphology (Figure 6(b)).

**Figure 7:**
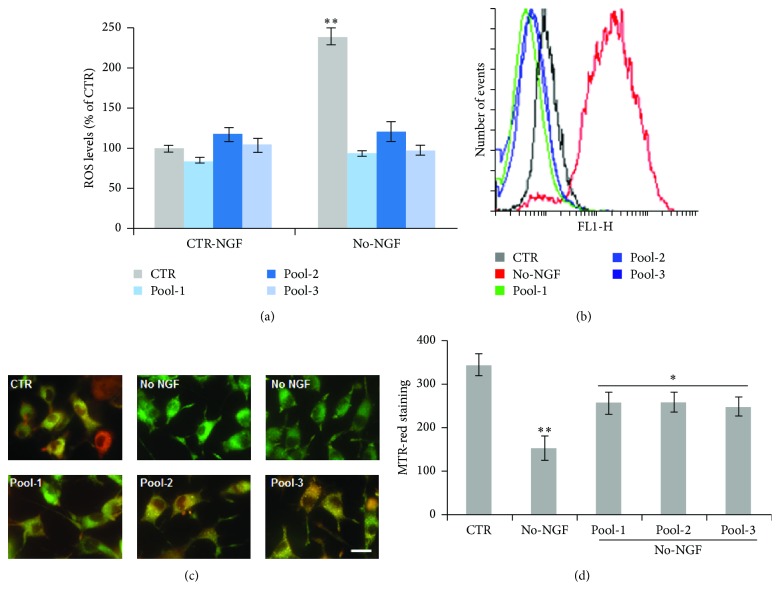
Effect of antioxidant cocktails on ROS levels and mitochondrial function following NGF deprivation. (a) Quantitation of ROS levels by FACS analysis of DCHF-DA fluorescence. Neuronal PC12 cells were preincubated overnight with Pool-1, Pool-2, or Pool-3 and then exposed to NGF-free medium for 6 hr. Cells were loaded with DCFH-DA (10 *μ*M) during the last 30 min of treatments and flow cytometric measurements (Geo-mean values) were taken on 10,000 cells contained in the gated regions. Data are the mean ± SEM of three experiments in duplicate. ^*∗∗*^ *p* ≤ 0.01 versus CTR-NGF (ANOVA and Dunnett's multiple comparisons test). (b) Overlapping FACS profiles of a representative experiment. (c) Representative merged images of neuronal PC12 cells maintained in the presence of NGF (CTR) or NGF-deprived for 12 h. NGF-differentiated PC12 cells were pretreated overnight with Pool-1, Pool-2, or Pool-3 before NGF withdrawal in the presence of the indicated pools. Cells were stained by MitoTracker Red and Green (50 and 200 nM, resp.) during the last 30 min of treatment and observed with a fluorescence microscope (Nikon) equipped with a CCD camera. Images were captured at 60x magnification. Scale bar = 10 *μ*m. (d) MitoTracker Red fluorescence was quantified by NIH ImageJ, and the MFI was calculated by counting all cells (on average 200 cells) in about 20 random fields for each condition. Data, expressed as MFI, are the mean ± SEM of three experiments in duplicate. ^*∗*^ *p* ≤ 0.05 versus No-NGF, ^*∗∗*^ *p* ≤ 0.01 versus CTR (ANOVA and Dunnett's multiple comparisons test).

To assess whether low doses of antioxidant cocktails were endowed with appropriate redox potential, we measured intracellular ROS levels.  Figure 7 shows that pretreatment of neuronal PC12 cells with Pool-1 partially decreased basal ROS levels and fully prevented the strong increase of ROS production induced by NGF withdrawal (Figures 7(a)-7(b)). A significant reduction of intracellular ROS reduction was also observed when cells were preincubated overnight with Pool-2 and Pool-3 before NGF deprivation for 6 h (Figures 7(a)-7(b)), as compared to single treatments at the concentrations used in the cocktails (data not shown). Moreover, overnight preincubation of neuronal PC12 cells with Pool-1, Pool-2, and Pool-3 prevented the decrease of mitochondrial potential induced by NGF withdrawal for 12 h (Figures 7(c)-7(d)), as compared to single treatments at the concentration used in the cocktails (data not shown). All together, these data suggest that supplementation of antioxidant cocktails at low-doses might have beneficial effects similar to those achieved with single treatments at higher doses, by preserving mitochondrial function and cellular homeostasis.

## 4. Discussion

Loss of cholinergic neurons resulting from reduced NGF signaling is believed to underlie the onset of sporadic AD [7]. Although the complex molecular mechanisms leading to apoptotic neuronal death have been extensively detailed [5, 7], it is crucial to identify new means for effective neuroprotection. We provide new evidence about the efficacy of a number of dietary antioxidants in restoring cell viability under conditions of NGF deprivation by decreasing oxidative stress and mitochondrial dysfunction. To examine the activity of these molecules, we used NGF-differentiated PC12 cells, which are widely used to investigate molecular events linked to NGF deprivation and A*β* toxicity [10, 44], as well as to study intracellular signaling induced by oxidative stress or neurotoxins involved in PD [4, 14, 45, 46].

Although it is still not clear whether oxidative stress is the initiating event associated with neurodegeneration, several data indicate that it is common to all neurodegenerative conditions. It is known that most dietary supplements, that is, polyphenols, carotenoids, thiolic compounds, vitamins (vit. C and vit. E), and selenium among oligoelements, are potent antioxidants able to reduce the oxidative stress due to increased ROS production. Indeed, the antioxidant activity of all dietary factors tested in this work has been demonstrated in different cellular and animal models of neurodegenerative conditions linked to oxidative stress, such as those involved in AD (A*β* and tau toxicity), PD (*α*-synuclein, 6-hydroxydopamine and MPTP), MS, and ischemia (oxygen-glucose-deprivation) [41, 42, 44, 47]. At the same time, all these studies have elucidated a number of molecular mechanisms that involve activation of sirtuins, transcription factors (in particular, NF-*κ*B and Nrf2 and PPAR/PGC-1*α*), and pathways that regulate metabolism, antioxidant responses, and cellular homeostasis [16, 24–26].

Although a few reports have shown that RSV and QRC potentiate NGF activity on neurite outgrowth [48–50], very little is currently known about the activity of dietary supplements under conditions of reduced neurotrophic support, such as decreased NGF availability, which is known to be associated to age-related neurodegeneration, in particular AD [5, 7]. Therefore, we used neuronal NGF-dependent PC12 cells as a model to evaluate whether oxidative stress and decreased survival due NGF deprivation can be counteracted by dietary antioxidants.

It is known that NGF promotes the survival of target neurons through the activation of the PI3K-Akt signalling. This pathway plays a key role against oxidative damage by regulating the antioxidant machinery including Cu/Zn-SOD, the stress response protein heme oxygenase-1 (HO-1), catalase, and glutathione peroxidase activities [13–15]. Through PI3K/Akt and NF-*κ*B, NGF also regulates the mitochondrial function by modulating levels of bcl-2 family proteins [12]. In agreement with these findings, we showed that NGF withdrawal of neuronal PC12 cells for 24 h resulted in a significant reduction of cell viability (Figure 1) that was preceded by enhanced ROS production at 6 h (Figure 4) and decreased ΔΨ*m* at 12 h (Figure 5).

Based on knowledge that all antioxidant molecules have distinct modes of function and complementary action on transcription factors, nuclear receptors, and enzymes, in this exploratory study, we analyzed the activity of a number of dietary antioxidants that have been found to be effective in other models of neuronal injury. We newly discovered that neuronal PC12 cells death induced by NGF deprivation was significantly prevented by overnight preincubation of NGF-dependent PC12 cells with RSV 10 *μ*M (Figure 2(a)), QRC 10 *μ*M (Figure 2(b)), GTE 12.5 *μ*g/mL (Figure 2(c)), OLP 10 *μ*g/mL (Figure 2(d)), CRC 10 *μ*M (Figure 2(e)), LYC 5 *μ*M (Figure 2(f)), NAC 300 *μ*M (Figure 2(g)), ALA 10 *μ*M (Figure 2(h)), ALCAR 10–50 *μ*M (Figure 2(i)), CoQ 100 nM (Figure 2(j)), or Sel 50 nM (Figure 2(k)). For each molecule, we determined the lower concentrations that were useful in maintaining cell viability (Figure 2), while preserving their neuronal morphology (Figure 3) by reducing ROS levels (Figure 4) and restoring mitochondrial function (Figure 5).

The efficacy of all antioxidants was restricted to the low micromolar range (5–10 *μ*M), with the exception of NAC (30–300 *μ*M) and CoQ and Sel, which were beneficial at nanomolar concentrations (Figure 2). Lower concentrations were unable to prevent neuronal loss, while higher doses were found to be toxic. Emblematic was the case of RSV, which was effective only at the dose of 10 *μ*M, in agreement with previous studies [42]. Indeed, RSV has been reported to have a typical hormesis effect on a wide variety of cells [23]: it is commonly accepted that RSV has neuroprotective activity [25]; however, depending on its concentration, it can also induce apoptosis, proliferation, and cell migration of several tumor cell lines by activating several molecular effectors [23, 25, 27, 28]. A similar trend was observed for most dietary molecules (QRC, OLP, CRC, LYC, ALA, ALCAR, CoQ, and Sel) under conditions of NGF deprivation (Figure 2). Moreover, high concentrations of RSV, OLP, CRC, ALA, CoQ, and Sel decreased cell survival even under basal conditions (CTR-NGF; Figure 2).

Antioxidants, however, are usually taken up with the diet as a mixture from different foods, where they are present in limited amounts and absorbed at even lower amounts. To this purpose, this study aimed to also explore the activity of defined low-dose cocktails whose components have been selected on the basis of (i) their different structure [29] and (ii) complementary action on transcription factors, nuclear receptors, and enzymes [16, 25].

Interestingly, we found that distinct cocktails of selected antioxidants at low concentrations were very effective in sustaining viability of neuronal PC12 cells after NGF withdrawal (Figure 6). Similar levels of neuroprotection were observed with Pool-1 (RSV 2 *μ*M, QRC 2 *μ*M, NAC 5 *μ*M, and OLP 1 *μ*g/mL) and Pool-2 (RSV 1 *μ*M, QRC 1 *μ*M, NAC 10 *μ*M, OLP 1 *μ*g/mL, CRC 1 *μ*M, and LYC 1 *μ*M) (Figure 6(a)), suggesting that neuronal survival could be efficiently preserved by mixtures of molecules belonging to distinct classes of dietary antioxidants (flavonoids, nonflavonoids, carotenoids, and thiolic compounds) at concentrations lower than effective doses of single treatments (Figure 2). Cell viability was also maintained by Pool-3 (RSV 2.5 *μ*M, ALA 2.5 *μ*M, and ALCAR 2.5 *μ*M), which was the simplest cocktail giving the best neuroprotective activity (Figure 6(a)). All antioxidant mixtures, Pool-1, Pool-2, and Pool-3, also significantly reduced ROS levels and prevented mitochondrial dysfunction (Figure 7), while maintaining the neuronal morphology (Figure 6(b)).

Although cooperation of two or more antioxidants have been previously reported both* in vitro* and* in vivo* [41, 51, 52], to our knowledge synergistic protection by low doses of antioxidant cocktails has never been described so far. The efficacy of these mixtures could be attributed to the complementary action of dietary antioxidants of the pools on different enzymes and transcription factors that modulate metabolism and mitochondrial function.

Supplementation of one single dietary factor, even at low effective concentrations, cannot be as much effective as a mixture, because its mechanisms of action are specific and limited; therefore it cannot efficiently restore cellular homeostasis. Moreover, it is now established that antioxidants have pleiotropic properties and additional biological functions going far beyond their antioxidant activity [16]. On the one hand, polyphenols and other dietary antioxidants can downregulate the synthesis of proinflammatory molecules in the course of inflammatory processes; on the other hand, they can stimulate the activity of resting cells, but a persistent stimulation can induce apoptosis of healthy cells. Therefore, studying the activity of low doses of cocktails might be relevant for future studies about the administration of mixtures of dietary factors under more physiological conditions.

In conclusion, our data (i) provide new evidence on the role of a number of dietary supplements in promoting survival of neuronal PC12 cells following NGF deprivation and (ii) identify some low-dose antioxidant cocktails that might better afford neuroprotection against oxidative stress and neuronal dysfunction under more physiological conditions. Further studies are in progress to elucidate the complementary role of the various antioxidants of the pools in modulating enzymes and transcription factors that modulate metabolism and mitochondrial function.

## Figures and Tables

**Table 1:** Concentrations of antioxidants in the cocktails.

	RSV	QRC	NAC	OLP	CRC	LYC	ALA	ALCAR
Pool-1	2 *μ*M	2 *μ*M	5 *μ*M	1 *μ*g/mL				
Pool-2	1 *μ*M	1 *μ*M	10 *μ*M	1 *μ*g/mL	1 *μ*M	1 *μ*M		
Pool-3	2.5 *μ*M						2.5 *μ*M	2.5 *μ*M

## Abbreviation

NGF: Nerve Growth Factor
ROS: Reactive oxygen species
DCHF-DA: 2′,7′-Dichlorodihydrofluorescein-diacetate
RSV: Resveratrol
QRC: Quercetin
CRC: Curcumin
LYC: Lycopene
ALA: Alpha-lipoic acid
OLP: Oliplus
GTE: Green tea extract
NAC: N-Acetylcysteine
ALCAR: Acetyl-L-Carnitine
CoQ: Coenzyme Q10
Sel: Selenium.
